# Current development of bovine jugular vein conduit for right ventricular outflow tract reconstruction

**DOI:** 10.3389/fbioe.2022.920152

**Published:** 2022-08-04

**Authors:** Chenggang Li, Bo Xie, Ruizhe Tan, Lijin Liang, Zhaoxiang Peng, Qi Chen

**Affiliations:** ^1^ Xuzhou Third People’s Hospital, Xuzhou, Jiangsu, China; ^2^ Renji Hospital, Shanghai Jiao Tong University School of Medicine, Shanghai, China; ^3^ Ningbo Regen Biotech, Co., Ltd., Ningbo, Zhejiang, China; ^4^ The Affiliated Lihuili Hospital, Ningbo University, Ningbo, Zhejiang, China

**Keywords:** right ventricular outflow tract (RVOT), bovine jugular vein conduit (BJVC), aneurysmal dilatation, infective endocarditis, stenosis, crosslinking, decellularizalion, bioreactor

## Abstract

Right ventricular outflow tract (RVOT) reconstruction is a common surgical method to treat congenital cardiac lesions, and bovine jugular vein conduit (BJVC) has become a prevalent candidate of prosthetic material for this procedure since 1999. Although many clinical studies have shown encouraging results on BJVCs, complications such as stenosis, aneurysmal dilatation, valve insufficiency, and infective endocarditis revealed in other clinical outcomes still remain problematic. This review describes the underlying mechanisms causing respective complications, and summarizes the current technological development that may address those causative factors. Novel crosslinking agents, decellularization techniques, conduit coatings, and physical reinforcement materials have improved the performances of BJVCs. The authors expect that the breakthroughs in the clinical application of BJVC may come from new genetic research findings and advanced characterization apparatuses and bioreactors, and are optimistic that the BJVC will in the future provide sophisticated therapies for next-generation RVOT reconstruction.

## Introduction

Reconstruction of right ventricular outflow tract (RVOT) is a common method of surgical correction for various congenital cardiac lesions, including pulmonary atresia or stenosis, truncus arteriosus, tetralogy of Fallot, transposition of great arteries, and double outlet right ventricle (Ruzmetov et al., 2012). This type of surgery has been made possible with a valved conduit, which was firstly an allograft aortic valve conduit in 1966 (Ross, 1966), and later took various forms such as porcine pulmonary valve conduit, bovine jugular vein conduit, valved bovine/porcine pericardial tube, and valved synthetic tube (Ruzmetov et al., 2012; Salem, 2016).

Bovine jugular vein conduit (BJVC) contains naturally integrated valves, and is easily available in large quantities with a range of sizes that can match the needs of both pediatric and adult patients. Therefore, since its successful commercialization (Contegra^®^, Medtronic Inc.) and introduction into clinical practice in 1999, BJVC has become the favored alternative to allografts for RVOT reconstruction in many hospitals (Rastan et al., 2006; Baslaim, 2008). To date, a considerable amount of studies have reported on the BJVCs. It appears that while some surgeons are satisfied with the conduits’ performances, others have certain concerns (Protopapas and Athanasiou, 2008). The majority of the publications regarding BJVCs, however, paid more attention to the clinical outcomes than the underlying mechanisms of conduit failures and complications, or the conduits’ innate properties. Therefore, this review will take a biomaterials science point of view and attempt to link clinical findings of BJVCs to relevant biochemical and biophysical researches, so that potential aspects of improvement could be made aware to peer researchers for developing a more durable BJVC.

## Current BJVCs and their clinical outcomes

Table 1 lists representative clinical studies on BJVCs used for RVOT reconstructions. First of all, by directly comparing the researchers’ verdicts, we can see a lack of consensus on the performances of the BJVCs. This is probably because there are a myriad of contributing factors affecting the clinical outcomes, such as demographic characteristics of patients, surgical techniques, and batch difference of products, which could all vary from center to center. Particularly, as more and more studies reached the long-term phase in the past 5 years, many latent risk factors (or the absence of them) truly started to show significant influences on the outcomes. Additionally, the choice of BJVCs was no longer limited to Contegra, the newly commercialized Balance Medical BJVCs, as well as individual centers’ homemade BJVCs, could all add variables to the clinical studies. The mortality rates in all the studies were deemed acceptable, and in most of the cases deaths were not conduit-related. Therefore, our current task is to improve the durability of the BJVC (i.e. to reduce the rates of explantation, reintervention, infection, etc.), and prolong the life of BJVC in RVOT reconstruction (Qian et al., 2021a).

**TABLE 1:** Clinical studies on bovine jugular vein conduits for right ventricular outflow tract reconstruction.

Choice of BJVC	Center	Data collected between	Time of follow-up	Population	Outcome	General Remarks On BJVC	References
Contegra	James Whitcomb Riley Children’s Hospital at Indiana University, and Cardinal Glennon Children’s Hospital at Saint Louis University, United States	January 1999 - August 2010	Mean: 48.4 ± 31 months Range: 1 month - 11 years	232	Early death: 4Late death: 8Explant: 24	An excellent immediate substitute for right ventricular outflow tract reconstruction	Fiore et al. (2011)
						
Contegra	Indiana University School of Medicine, United States	1999–2016	Mean: 4.0 ± 4.2, 4.9 ± 4.2, 5.9 ± 4.1 years depending on age group	276	Early death: 7	A useful option for right ventricular outflow tract reconstruction	Patel et al. (2018)
					Late death: 7		
					10-years freedom from explantation: 59%		
Contegra	Alder Hey Children’s Hospital, UK	October 1999 - February 2009	Mean: 4.6 ± 2.3 years Range: 8 months - 10 years	198	Early death: 5	A reliable alternative to pulmonary homografts	Prior et al. (2011)
					Late death: 5		
					10-years freedom from conduit failure: 90%		
Contegra	Royal Children’s Hospital, Australia	2001–2011	Mean: 5 ± 3.2 years Range: 1 month - 11.9 years	113	Early death: 5	Contegra conduits and homografts have comparable mid-term outcomes	Yong et al. (2015)
					Late death: 5		
					5-years freedom from conduit replacement: 75%		
Balance Medical BJVC	Children’s Hospital of Fudan University, China	January 2002 - December 2013	Median: 6.3 years Interquartile range: 4.9–8.6 years	53	Early death: 2	The durability of BJVC is suboptimal after a mid-term follow-up period	Zhang et al. (2017)
					Late death: 0		
					7-years freedom from conduit failure: 62.1%		
Balance Medical BJVC	Shanghai Children’s Medical Center, China	2009–2018	Median: 33.3 months Range: 3.3 months -10.1 years	102	Early death: 9	BJVCs have acceptable mid-term Outcomes	Chen et al. (2019)
					Late death: 3		
					5-years freedom from reintervention: 60.9%		
Contegra	Texas Children’s Hospital, United States	2001–2017	Median: 6 years Range: 5 months -14 years	228	Early death: 2	This high incidence of late endocarditis is concerning and warrants intervention	Beckerman et al. (2018)
					Late death: 4		
					5-years freedom fmm replacement: 84%		
					10-years freedom from replacement: 49%		
Homemade BJVC	National Center for Cardiovascular Diseases and Fuwai Hospital, China	December 2003 - January 2016	Median: 37.2 months Range:0.4–129.2 months	10	Early death: 2	The use of a BJVC was a main reason for right ventricle-pulmonary artery restenosis	Wang et al. (2018)
					Late death: 0		
					None underwent reoperation		
					5/10 had higher gradients (over 40 mmHg)		
Contegra	Heart and Diabetes Center North- Rhine Westphalia and Hannover Medical School, Germany	1999–2012	Mean:4.3 ± 3.8 years	444	Freedom from explantation after 12 years: 75%	The use of bovine jugular veins for RVOT in patients younger than 25 years leads to superior results compared with cryopreserved homografts	Sandica et al. (2016)
Contegra	Colorado Children’s Hospital, United States	January 2009 -December 2017	Median: 3.6 years	109	4 deaths at 0.1, 0.3, 0.4, and 3.8 years post implant. At 12months 28% of unsupported BJVCs had moderate to severe regurgitation versus 4% of supported BJVCs	There is a relatively high incidence of endocarditis	Lueth et al. (2019)
Contegra	Mazankowski Alberta Heart Institute, Canada	January 2000 - August 2012	Median: 3.2 years Range: 2 days - 11.7 years	244	Early death: 2	BJVC was associated with a significantly higher incidence of bacterial endocarditis and conduit deterioration in older children	Ugaki et al. (2015)
					Late death: 15		
					7-years freedom from conduit replacement: 64.2%		
					10-years freedom from conduit replacement: 37.1%		
Contegra	National Cerebral and Cardiovascular Center, Japan	April 2013 - April 2014	Mean: 10 months Range: 5–18 months	13	2 deaths at 1.3 and 2months	Indication for using BJVC should be carefully considered	Kido et al. (2016)
					10-months freedom from reintervention: 53%		
Contegra	Okayama University Hospital, Japan	January 2013 - December 2017	Mean: 4.9 ± 1.9 years	20	Death: 0	Outcomes of RVOT reconstructions with BJVCs were clinically satisfactory	Hirai et al. (2021)
					Freedom from replacement in follow-up period: 80%		
Contegra	A center in Jeddah, KSA	2002–2011	Mean: 19.8 months Range: 8–78 months	22	Early death:1	A good conduit for RVOT reconstruction	Sersar et al. (2014)
					Late death: 0		
Contegra	5 institutes in Japan	April 2013 -December 2019	Median: 3.1 years Range: 13–5.1 years	178	Death: 15 5-years conduit explantation-free survival rate: 71.0%	Mid-term outcomes of RVOT reconstruction with BJVCs were acceptable	Hoashi et al. (2021)

* Early death is defined as a death in the hospital or within 30 days of discharge. All other events are considered late (Brown et al., 2006).

## Complications and causes

Complications accounted for the majority of conduit failures. By finding out the underlying mechanisms of each complication, we may strive to eliminate risk factors in conduit design and manufacture, and therefore increase the long-term rate of freedom from conduit failure.

### Stenosis

Conduit stenosis is the most common complication after RVOT reconstruction with BJVC, and often requires reintervention (Fiore et al., 2011; Boethig et al., 2012). In contrast to allografts and porcine xenografts where stenoses are mainly resultant from conduit wall calcification (Hellberg et al., 1981; Dittrich et al., 2000; Tweddell et al., 2000; Forbess et al., 2001; Wells et al., 2002), BJVCs, although not totally free from calcification, seem to be less susceptible to this risk (Meyns et al., 2004; Fiore et al., 2011). Nonetheless, there has still been a non-negligible incidence of reintervention for BJVCs, in order to relieve stenoses caused by neointimal hyperplasia (Meyns et al., 2004; Kido et al., 2016; Nichay et al., 2018).

Given its known cytotoxicity, the crosslinker glutaraldehyde is again the usual suspect held responsible for neointimal hyperplasia. Brown et al. and Boethig et al. believed residual glutaraldehyde could trigger the development of neointimal fibrosis and subsequent stenosis, and therefore recommended thorough rinsing of the BJVCs before implantation (Brown et al., 2006; Boethig et al., 2012). Chang et al. reported in a canine model that glutaraldehyde might hinder re-endothelialization of the BJVC, which could contribute to inflammatory reaction and neointimal proliferation (Chang et al., 2001). It has indeed been established that there is an association between glutaraldehyde treatment and tissue calcification (Tod and Dove, 2016), and the cellular mechanisms are relatively well understood (calcium influx into devitalized but phosphorus-rich cells) (Kim, 2001; Schoen and Levy, 2005). However, as for the role that glutaraldehyde may have played in inducing neointimal hyperplasia, very little is known.

Unsatisfied with accepting glutaraldehyde as an ambiguous answer, researchers keep seeking for other causal mechanisms of neointimal hyperplasia. It has been reported in many studies that stenoses occur more frequently at the distal conduit anastomosis than at the proximal anastomosis (Kadner et al., 2004; Boethig et al., 2005; Kido et al., 2016). Attentions are therefore paid to this particular region. First of all, there exist discrepancies in the circumference or diameter between the conduit and the native pulmonary bifurcation, as well as conduit kinking at the distal anastomosis due to its occasionally excessive length. These are stenosis risk factors on their own as they lead to crowding of the conduit wall tissue (Meyns et al., 2004; Kido et al., 2016). But what is more important is that, the resultant blood flow abnormality is believed to have induced repetitive trauma to the neointima, causing an excessive neointimal proliferation, shown as the stenotic fibrotic tissue at the distal anastomosis (Kadner et al., 2004; Meyns et al., 2004; Peivandi et al., 2019).

Apart from the aforementioned causes of stenosis, the potential host immunologic reaction to the BJVC should never be overlooked. Lacour-Gayet pointed out that, as a xenogenic tissue, despite the treatments in the manufacture process, the BJVC may still contain active class I and II major histocompatibility complex (MHC) residual antigens (Brown et al., 2006). Wojtalik et al. were able to observe a significant rise in B cells between the third and sixth month postoperatively. The T-lymphocyte activation study also revealed higher number of CD69^+^ and CD71^+^ cells 1 year after implantation (Wojtalik et al., 2003). These immune responses may lead to not only stenosis at the distal anastomosis but also intensive narrowing along the whole length of the conduit (Göber et al., 2005).

What’s worth noticing is that, young age of patient, and the associated small size (≤14 mm in diameter) of the received conduit are clinically considered an independent risk factor for stenosis (Fiore et al., 2010; Gist et al., 2012; Zhang et al., 2017). But in the authors’ opinion, small conduit size does not intrinsically cause stenosis or other complications. Neither is over-sizing the conduit necessarily advantageous (Karamlou et al., 2005; Karamlou et al., 2006). It is more essentially the *in vivo* behaviours of the BJV material, for example the decreased luminal size due to neointimal hyperplasia and lack of somatic growth (Pennel and Zilla, 2020; Hoashi et al., 2021), that make it more prone to cause problems in small conduits.

### Aneurysmal dilatation and valve insufficiency

Aneurysmal dilatation is considered to be associated with the distal anastomotic stenosis, and is usually found at the proximal anastomosis (Yoldaş et al., 2019). The stenosis-induced high right ventricle (RV) and intra-conduit pressure casts increased stress to the conduit wall, and the venous tissue (expectedly not as elastic as the arterial tissue) is irreversibly deformed and dilated (Delmo-Walter et al., 2007). This is more likely to occur in neonates and young infants, because the small size of the conduits they receive are more conducive to the high RV and intra-conduit pressure, and their hypoplastic pulmonary arteries are also more susceptible (Boudjemline et al., 2003a).

The dilatation, if close to the valve section, will then potentially result in valve insufficiency (Boudjemline et al., 2003b). As the leaflets in BJVC have excess tissue and can tolerate certain dilatation without competence loss (Boudjemline et al., 2003a), in some patients the valve insufficiency is mild or even not seen, and therefore clinical reintervention is not required. If progressed, however, the dilatation will almost certainly lead to significant regurgitation, and these patients will then have to undergo reoperations (Brown et al., 2006; Morales et al., 2006; Shebani et al., 2006).

In some rare cases, dilatation could occur in the absence of stenosis or raised pressure, and could not be explained by the above-mentioned mechanism. Yoldaş et al. reported this type of “true aneurysmal dilatation”, in which the explanted conduit was uniformly dilated from the proximal anastomosis to the distal anastomosis, indeed different from common findings (Yoldaş et al., 2019). Although further evidence should be drawn from more conduits of this sort when they come up for replacement, the authors speculate that the prevalent degeneration of elastic fibers in the conduit may be responsible, as it has been reported to have led to reduced tissue elasticity and flow abnormalities (Peivandi et al., 2019).

### Infective endocarditis

Infective endocarditis (IE) is another concerning postoperative complication in BJVC implantation. It seems to have a tendency to occur late (Albanesi et al., 2014; Mery et al., 2016), and therefore is raising more awareness in recent years among the clinicians as the long-term outcomes start to be revealed. What is of greater importance, however, is that BJVCs are reported to have significantly higher risks of IE compared with other valved conduits (Malekzadeh-Milani et al., 2014; Ugaki et al., 2015), the understanding of which may be critical in making future surgical options (Haas et al., 2018).

Researchers have so far established that the infection is unlikely to have resulted from procedural contamination or tissue-borne bacteria (Malekzadeh-Milani et al., 2014), but is associated with daily predisposing factors such as dental and skin problems (Yoshinaga et al., 2008; Knirsch and Nadal, 2011; Bos et al., 2021). As for the specific propensity of BJVCs to IE, altered flow patterns, immune responses, and abnormal surface conditions (e.g. traumatized endothelium) were firstly proposed to be the contributing factors (Ugaki et al., 2015; Mery et al., 2016; Sharma et al., 2017). These causes were plausible, because all of them could partially explain the neointimal hyperplasia at the same time. However, more detailed and convincing pathophysiologic mechanisms remained unclear. Jalal et al. and Veloso et al. therefore made pioneering attempts to tackle this issue from the angle of tissue surfaces’ susceptibility to bacterial adherence (Jalal et al., 2015; Veloso et al., 2018a). The former research group reported a higher bacterial adhesion on bovine jugular vein (BJV) tissue than on bovine and porcine pericardium, while the latter compared BJV tissue, bovine pericardium and cryopreserved allograft, and found bacterial adhesion to be tissue-insensitive. Both studies were conducted *in vitro*, and despite the somewhat incongruous results, they provided valuable insights into a future research direction on this specific topic, which was the interaction between the BJV tissue and plasma proteins, and the subsequent interaction with blood-borne bacteria (Jalal et al., 2018; Mery, 2018). In fact, in a follow-up study by Veloso et al., BJV tissue did show greater absorption of fibrinogen (known to facilitate the adhesion of IE-associated pathogens) than cryopreserved allograft tissue (Veloso et al., 2018b).

## Modified treatments and designs

To address the complications raised above, with either confirmed or speculated causes, researchers have proposed various modified treatments in conduit processing and new conduit designs. These modifications focused on the perspectives of mechanical properties, immunogenicity, calcification resistance, infection resistance, and endothelialization ability, respectively.

### Crosslinking techniques

Ever since the clinical introduction of BJVCs, researchers have been seeking for alternative crosslinking agents to the conventional aldehyde-based agents, in order to minimize biological risks.

Noishiki et al. evaluated polyepoxy compound (ethylene glycol diglycydyl ether, glycerol polyglycidyl ether, etc.) as the crosslinker on vascular grafts in the 1980s and ‘90s, and found that compared with glutaraldehyde, polyepoxy compound reduced the level of calcification and retained the natural elasticity and appearance of the biological materials (Tomizawa et al., 1988; Hata et al., 1992; Noishiki et al., 1993). This series of studies has recently been furthered by Nichay et al. in biochemical and biophysical settings (Zhuravleva et al., 2018; Zhuravleva et al., 2022). They quantitatively reported the calcium accumulation in the polyepoxy-treated BJV, which was 25% of that in the glutaraldehyde-treated experimental group, and 40% of that in the commercially available Contegra (also glutaraldehyde-treated). They also found that polyepoxy-treated samples had a higher degree of hydration than both fresh BJV samples and glutaraldehyde-treated samples. Hydration also lead to lower mechanical properties (stiffness and failure strength) of the polyepoxy-treated samples compared with their glutaraldehyde-treated counterparts. These mechanical characteristics, however, as Nichay et al. pointed out, was not necessarily inferior, because they were actually more close to the mechanical properties of a native human pulmonary artery (Zhuravleva et al., 2022).

Wu and his team have been taking another crosslinking approach, dye (methylene blue)-mediated photooxidation since 2004 (Feng et al., 2004). They compared photooxidatively crosslinked BJVCs with glutaraldehyde-treated and polyepoxy-treated ones, and suggested that, while all of the conduits were cellular and humoral immunogenic, the photooxidatively crosslinked BJVCs had significantly lower level of immunogenicity (Wang et al., 2005). They have also recently reported positive midterm clinical outcomes of photooxidatively crosslinked acellular BJVCs. It was shown that dye-mediated photooxidation, combined with decellularization technique (which will be discussed below), helped the BJVCs to achieve resistance to calcification and infection, and to exhibit appropriate dilation with age (Qian et al., 2021b).

Other options of crosslinking agents include naturally occuring chemicals, which are reasonably considered to be more biocompatible. Xu et al. investigated proanthocyanidin *in vitro* and found it to significantly reduce risk of hemolysis in BJVCs while generating similar resistance to collagenase degradation, compared with glutaraldehyde (Xu et al., 2012). Chang et al. conducted an *in vivo* study of genipin-fixed BJVs, and highlighted their superior endothelialization to the glutaraldehyde-fixed BJVs, as well as minimal inflammatory reaction and intact valvular leaflets after 6 months (Chang et al., 2001).

Last but not least, the traditional glutaraldehyde never actually went off researchers’ radar. Appropriate additives can suppress the cytotoxicity of glutaraldehyde and re-expand its biomedical applications. The most common co-crosslinking agents of glutaraldehyde are amino acids (e.g. glycine, glutamic acid, l-lysine, and taurine), which react with and hence block free aldehyde groups (Bezuidenhout et al., 2009; Braile et al., 2011; Park et al., 2017; Meuris et al., 2018; Braile-Sternieri et al., 2020). Jiang et al. recently investigated a tripeptide l-glutathione (composed of cysteine, glutamic acid, and glycine) and found that it yields even more outstanding cytocompatibility than glycine (Jiang et al., 2022). Apart from amino acids and peptides, Zhuravleva et al. assessed glutaraldehyde-treated BJV tissues modified with bisphosphonates. Both of the two bisphosphonates (pamidronic acid and 2-(2’ -carboxyethylamino)ethylidene-1,1-bisphosphonic acid) they studied were satisfactorily immobilized on the BJV tissue, and exhibited calcification-inhibitory effects through their ability to block nucleation and prevent the growth of hydroxyapatite crystals (Zhuravleva et al., 2021). Ding et al. took an even more sophisticated recipe, firstly introducing 2-amino-4-pentenoic acid as a co-crosslinker with carbon-carbon double bonds and then grafting on poly (ethylene glycol) diacrylate by radical polymerization, which not only resulted in better cytocompatibility but also improved hemodynamic property (Ding et al., 2022).

### Decellularization

Decellularization, as a key technique to reduce the immunogenicity of non-autologous biomaterials, was already employed in vascular prostheses in the 1980s (Malone et al., 1984), and it has also been incorporated into the conduit preparation process in most of the recent studies on BJVCs. The early decellularization approaches were based on detergents (Triton X-100, Tween, sodium dodecylsulphate, sodium deoxycholate, sodium cholate, etc.) and enzymes (trypsin, DNase, RNase, etc.), which induced lysis and removal of cells (Naso and Gandaglia, 2018). Lu et al. showed that the detergent-enzymatic decellularization technique to some extent compromised the extracellular matrix (ECM) microstructure of the BJV tissue, as well as its thermal stability and mechanical property (Lu et al., 2007). This would again suggest that the appropriate crosslinking techniques discussed above were indispensable, for counteracting the effects of decellularization.

At the same time, researchers were also endeavouring to reduce the usage of chemical agents that could denature ECM proteins (such as sodium dodecylsulphate) in the decellularization procedure (Keane et al., 2015). Sawada et al. reported using supercritical carbon dioxide (scCO2) as an extraction medium to remove the cells from porcine aorta. The obtained acellular tissue showed no decrease in mechanical strength (Sawada et al., 2008). Bakuleva and her team have recently modified this technique and applied it to BJV tissues (Chaschin et al., 2020; Chashchin et al., 2021). They developed a “hybrid” treatment which consisted of a short exposure to the standard detergent solution (1% v/v sodium dodecylsulphate) followed by an exposure to the scCO2 with Tween-80, in order to achieve complete decellularization and to preserve the ECM components (such as collagen and elastin) and structures.

A decellularized allograft pulmonary valve - CryoValve^®^ SG by CryoLife Inc., was introduced to clinical use in 2000, whereas the decellularized BJVC, as a xenograft counterpart, is still seeking FDA approval. In the mid-to long-term studies by Ruzmetov et al. and Bibevski et al., CryoValve^®^ SG demostrated in the RVOT reconstruction less insufficiency and stenosis, and hence better durability than the standard cryopreserved allograft (Ruzmetov et al., 2012; Bibevski et al., 2017). One would therefore analogically expect a superior performance from a decellularized BJVC than a standard BJVC (e.g. Contegra^®^). Yaxin Medical Technology Co., Ltd., (Wuhan, China.) is currently trialing its decellularized BJVC (in collaboration with Wu’s team) (Qian et al., 2021b), which has shown satisfactory durability and functionality so far, and bears the potential of becoming commercially available.

### Other modifications

In addition to the widely adopted crosslinking and decellularization techniques, researchers have also tried various bespoke coatings to optimize the performance of BJVCs in one or more aspects.

Chitosan was one of the favourable coating materials, in light of its known antimicrobial properties (Gallyamov et al., 2014). Chashchin et al. deposited chitosan from carbonic acid solution to decellularized BJVC, endowing the conduit a smooth intima surface and resistance to calcification. Meanwhile, the coating was also found to have significantly improved the strength and the elasticity of the decellularized conduit (Chaschin et al., 2020; Chashchin et al., 2021). Krasilnikova et al. applied globular chitosan, which had better diffusion and tissue-penetration characteristics than linear chitosan, to both cellular and acellular BJVCs. Subcutaneous implantation also demonstrated resistance to calcification. Mechanically, the treated acellular samples turned out to have greater strength and stiffness than the treated cellular samples, due to a higher level of impregnation of chitosan in the ECM (Krasilnikova et al., 2018). Moreover, Liu et al. trialed coatings of fibronectin, collagen Ⅳ and gelatin (Liu et al., 2009), while Tao et al. investigated nano-assemblies of heparin and dihydroxy-iron (Tao et al., 2012). Both treatments were for enhanced endothelialization in decellularized BJVCs, and they both generated positive results.

Guhathakurta et al. made particular efforts to counteract the aforementioned risks of dilatation, by electrospinning circumferentially oriented nanofibres onto the external surface of decellularized BJVC. The polymeric nanofibres (poly-caprolactone, poly-lactic acid and collagen type I) gave the BJVC extra resistance to dilatation while still keeping it biocompatible (Guhathakurta et al., 2011). In terms of physical reinforcement, the conduit manufacturers actually have more pragmatic solutions. The Contegra has been available in a ring-supported model, which has two external polypropylene rings sutured to the adventitial layer of the conduit intended for limiting dilatation at the valvular section (Boudjemline et al., 2003b; Morray et al., 2017), although no clinically significant echocardiographic or outcome differences seemed to have been found between the ring-supported and unsupported Contegras (Lueth et al., 2019). The Balance Medical BJVC also has a polyester film covering the outer wall of the vein to prevent dilatation, and the device has been preferred for older children with hypoplastic pulmonary arteries (Zou et al., 2021).

## Future prospects

The BJVC has undoubtedly become an important means of RVOT reconstruction, and will continue to be one in the future. Studies that compare BJVCs with allografts and synthetic polymer conduits will also be ongoing, and so is the debate as to which one of them is better. In the authors’ opinion, however, at least in the coming few years the focuses of research should still be placed on how to perfect each one of the treatment regimes, rather than conclusively making a choice from them.

Hence for BJVC, the goal of future research should include finding more biocompatible crosslinking agents, optimizing decellularization protocols and storage strategies, and developing more delicate conduit designs (Xiling et al., 2022). At the same time, the manufacturers of the commercialized BJVCs should strive to achieve the highest level of standardization in their products (which indeed is always a challenge for animal-derived biomaterials), and help clinical centers set up comprehensive databases. By plotting conduit-, patient-, and center-related variables against the outcomes, researchers can then better elucidate the causative factors of the occurring complications (Herrmann and Brown, 2020; Rapetto et al., 2021).

Apart from those, the authors expect that the breakthroughs in the clinical application of BJVC may come from two specific areas. The first one is genetic research, which identifies the patients’ predispositions to certain complications, or the bovine tissue’s susceptibility to certain immunologic reactions (Boudjemline et al., 2003c). For example, Senage et al. have very recently demonstrated that antibodies against the xenoantigens galactose-α1,3-galactose (αGal) and N-glycolylneuraminic acid (Neu5Gc) could mediate the deterioration of bioprosthetic heart valves through calcification, which was indeed a remarkable step forward (Senage et al., 2022). Furthermore, with the help of genetic engineering technologies, one may even achieve conduits that are deficient in certain antigen-expressing genes, or conduits that can produce human thrombo-regulatory and anti-inflammatory proteins (Yamamoto et al., 2015).

The other area is the development of *in vitro* characterization apparatuses and bioreactors. The biaxial tester might be considered one of the first few apparatuses that brought the research on BJV tissues to a higher level of sophistication. By linking the radial and circumferential mechanical properties of the BJV leaflets to their structural and compositional changes, the biaxial tests performed could provide insights into the pathogenesis of valve distension and incompetence (Huang and Lu, 2017; Benson and Huang, 2019). Rittgers et al. and Dur et al. constructed *in vitro* models simulating the physiological flows and pressures, and proposed certain parameters to measure (systolic-to-diastolic pressure drop (ΔP), resistance (R), effective orifice area (EOA), etc.) when testing the valves (Rittgers et al., 2007; Dur et al., 2010). Easson et al. developed a more straightforward testing system with a high-speed camera and an image analysis software. The captured valve behaviours (e.g. opening time and geometric orifice area) were able to reflect the property difference between various BJV tissues (such as fresh and glutaraldehyde-treated) (Easson et al., 2019). Fatigue property is one of the key indicators of the durability of a BJVC, and the ISO 5840 (2021) standard prescribes guidelines on fatigue assessment of heart valves (ISO, 2021). However, it does not specify the test methods or the test equipment. Numerous apparatuses have been built to test various forms of heart valves (Vesely et al., 1995; Butterfield and Fisher, 2000; Iwasaki et al., 2002; Arokiaraj et al., 2016; Raghav et al., 2016), but none of them is specifically designed for RVOT reconstruction conduits. A research group at University of Arkansas has recently been developing an accelerated valve fatigue apparatus for BJV tissues, and when completed it is going to offer quick assessment of the durability of a valved RVOT conduit (Brazhkina, 2019; Kueh, 2020). Bioreactors are generally the above dynamic perfusion systems supplemented with biological elements (such as cells, media with growth factors, or even bacteria) (Teebken et al., 2003; Ditkowski et al., 2019; Leeten et al., 2021). Compared with the characterization apparatuses, bioreactors hold even greater potentials. Ditkowski et al. and Leeten et al. used bioreactors to model BJVC behaviours - bacterial adherence and endothelialization, respectively (Ditkowski et al., 2019; Leeten et al., 2021), whereas many other researchers attempted to achieve cell repopulation of decellularized conduits with bioreactors (Teebken et al., 2003; Lichtenberg et al., 2006; Yuan et al., 2016; Iacobazzi et al., 2021; Rapetto et al., 2022). Although the development of an ideal bioreactor and a standard conduit-engineering protocol is still in it is infancy, a decellularized BJVC repopulated with autologous cells offering a close-to-autograft operation choice may well be where the future lies (Cleuziou, 2018).

## Summary

BJVC found an important place in reconstruction of RVOT, and clinical outcomes showed its competency as well as room for improvement. Complications such as stenosis, aneurysmal dilatation, valve insufficiency, and infective endocarditis still remain to be addressed. The BJVC can be modified by crosslinking, decellularization, coating, and various physical reinforcement techniques. Future researches may be pointed towards genetic studies and bioreactor designs. The authors optimistically expect that the BJVC will achieve significant improvement and provide sophisticated therapies for next-generation RVOT reconstruction.

## References

[B1] Albanesi F. Sekarski N. Lambrou D. Von Segesser L. K. Berdajs D. A. (2014). Incidence and risk factors for contegra graft infection following right ventricular outflow tract reconstruction: Long-term results. Eur. J. Cardio-Thoracic Surg. 45 (6), 1070–1074. 10.1093/ejcts/ezt579 24412830

[B2] Arokiaraj M. C. Centeno A. Pesquera A. Zurutuza A. (2016). Novel graphene-coated mechanical bi-leaflet valves after accelerated wear test of 40M test cycles in saline. Acta Cardiol. 71 (3), 331–347. 10.1080/ac.71.3.3152094 27594129

[B3] Baslaim G. (2008). Bovine valved xenograft (contegra) conduit in the extracardiac fontan procedure: The preliminary experience. J. Card. Surg. 23 (2), 146–149. 10.1111/j.1540-8191.2007.00524.x 18304129

[B4] Beckerman Z. De Leon L. E. Zea-Vera R. Mery C. M. Fraser C. D. (2018). High incidence of late infective endocarditis in bovine jugular vein valved conduits. J. Thorac. Cardiovasc. Surg. 156 (2), 728–734. e2. 10.1016/j.jtcvs.2018.03.156 29753513

[B5] Benson A. A. Huang H. Y. S. (2019). Tissue level mechanical properties and extracellular matrix investigation of the bovine jugular venous valve tissue. Bioengineering 6 (2), 45. 10.3390/bioengineering6020045 PMC6630446 31091689

[B6] Bezuidenhout D. Oosthuysen A. Human P. Weissenstein C. Zilla P. (2009). The effects of cross-link density and chemistry on the calcification potential of diamine-extended glutaraldehyde-fixed bioprosthetic heart-valve materials. Biotechnol. Appl. Biochem. 54 (3), 133–140. 10.1042/ba20090101 19882764

[B7] Bibevski S. Ruzmetov M. Fortuna R. S. Turrentine M. W. Brown J. W. Ohye R. G. (2017). Performance of SynerGraft decellularized pulmonary allografts compared with standard cryopreserved allografts: Results from multiinstitutional data. Ann. Thorac. Surg. 103 (3), 869–874. 10.1016/j.athoracsur.2016.07.068 27788940

[B8] Boethig D. Schreiber C. Hazekamp M. Blanz U. Pretre R. Asfour B. (2012). Risk factors for distal contegra stenosis: Results of a prospective European multicentre study. Thorac. Cardiovasc. Surg. 60 (03), 195–204. 10.1055/s-0031-1298062 22228091

[B9] Boethig D. Thies W. Hecker H. Breymann T. (2005). Mid term course after pediatric right ventricular outflow tract reconstruction: A comparison of homografts, porcine xenografts and Contegras. Eur. J. cardio-thoracic Surg. 27 (1), 58–66. 10.1016/j.ejcts.2004.09.009 15621472

[B10] Bos D. De Wolf D. Cools B. Eyskens B. Hubrechts J. Boshoff D. (2021). Infective endocarditis in patients after percutaneous pulmonary valve implantation with the stent-mounted bovine jugular vein valve: Clinical experience and evaluation of the modified Duke criteria. Int. J. Cardiol. 323, 40–46. 10.1016/j.ijcard.2020.08.058 32860844

[B11] Boudjemline Y. Beyler C. Bonnet D. Sidi D. (2003). Surprising outcome similarities between Contegra® bovine jugular vein conduit and shelhigh No-React® porcine pulmonary valve conduit: Role of immunologic reaction. Eur. J. Cardio-Thoracic Surg. 24 (5), 850–851. 10.1016/s1010-7940(03)00509-8 14583326

[B12] Boudjemline Y. Bonnet D. Agnoletti G. Vouhé P. (2003). Aneurysm of the right ventricular outflow following bovine valved venous conduit insertion. Eur. J. cardio-thoracic Surg. 23 (1), 122–124. 10.1016/s1010-7940(02)00660-7 12493522

[B13] Boudjemline Y. Bonnet D. Massih T. A. Agnoletti G. Iserin F. Jaubert F. (2003). Use of bovine jugular vein to reconstruct the right ventricular outflow tract: Early results. J. Thorac. Cardiovasc. Surg. 126 (2), 490–497. 10.1016/s0022-5223(03)00232-0 12928649

[B14] Braile M. C. V. B. Carnevalli N. C. Goissis G. Ramirez V. A. Braile D. M. (2011). *In vitro* properties and performance of glutaraldehyde-crosslinked bovine pericardial bioprostheses treated with glutamic acid. Artif. Organs 35 (5), 497–501. 10.1111/j.1525-1594.2011.01262.x 21595718

[B15] Braile-Sternieri M. C. Goissis G. Giglioti A. d. F. Ramirez V. D. A. Pereira N. P. (2020). *In vivo* evaluation of Vivere bovine pericardium valvular bioprosthesis with a new anti-calcifying treatment. Artif. Organs 44 (11), E482–E493. 10.1111/aor.13718 32364253

[B16] Brazhkina O. (2019). “Development of a model for accelerated fatigue testing in venous valves,” in Biomedical engineering (Fayetteville, Arkansas: University of Arkansas).

[B17] Brown J. W. Ruzmetov M. Rodefeld M. D. Vijay P. Darragh R. K. (2006). Valved bovine jugular vein conduits for right ventricular outflow tract reconstruction in children: An attractive alternative to pulmonary homograft. Ann. Thorac. Surg. 82 (3), 909–916. 10.1016/j.athoracsur.2006.03.008 16928507

[B18] Butterfield M. Fisher J. (2000). Fatigue analysis of clinical bioprosthetic heart valves manufactured using photooxidized bovine pericardium. J. Heart Valve Dis. 9 (1), 161–166. discussion 167. 10678391

[B19] Chang Y. Tsai C. C. Liang H. C. Sung H. W. (2001). Reconstruction of the right ventricular outflow tract with a bovine jugular vein graft fixed with a naturally occurring crosslinking agent (genipin) in a canine model. J. Thorac. Cardiovasc. Surg. 122 (6), 1208–1218. 10.1067/mtc.2001.117624 11726898

[B20] Chaschin I. S. Khugaev G. A. Krasheninnikov S. V. Petlenko A. A. Badun G. A. Chernysheva M. G. (2020). Bovine jugular vein valved conduit: A new hybrid method of devitalization and protection by chitosan-based coatings using super-and subrcritical СО2. J. Supercrit. Fluids 164, 104893. 10.1016/j.supflu.2020.104893

[B21] Chashchin I. S. Petlenko A. A. Zaitsev I. L. Bakuleva N. P. (2021). A study of resistance to calcification of bovine jugular veins with coatings deposited from solutions in carbonic acid. Dokl. Phys. Chem. 496, 8. 10.1134/S0012501621010012

[B22] Chen H. Shi G. Qiu L. Wang S. Xu Z. (2019). Outcomes of prosthetic valved conduits for right ventricular outflow tract reconstruction. Pediatr. Cardiol. 40 (4), 848–856. 10.1007/s00246-019-02081-8 30887063

[B23] Cleuziou J. (2018). Is there any progress in the search for the ideal conduit for reconstruction of the right ventricular outflow tract in young children? Transl. Pediatr. 7 (3), 226–228. 10.21037/tp.2018.06.02 30159249 PMC6087829

[B24] Delmo-Walter E. M. Alexi-Meskishvili V. Abdul-Khaliq H. Meyer R. Hetzer R. (2007). Aneurysmal dilatation of the Contegra bovine jugular vein conduit after reconstruction of the right ventricular outflow tract. Ann. Thorac. Surg. 83 (2), 682–684. 10.1016/j.athoracsur.2006.06.050 17258016

[B25] Ding K. Zheng C. Huang X. Zhang S. Li M. Lei Y. (2022). A PEGylation method of fabricating bioprosthetic heart valves based on glutaraldehyde and 2-amino-4-pentenoic acid co-crosslinking with improved antithrombogenicity and cytocompatibility. Acta Biomater. 144, 279–291. 10.1016/j.actbio.2022.03.026 35365404

[B26] Ditkowski B. Veloso T. R. Bezulska-Ditkowska M. Lubig A. Jockenhoevel S. Mela P. (2019). An *in vitro* model of a parallel-plate perfusion system to study bacterial adherence to graft tissues. J. Vis. Exp. 2019 (143), e58476. 10.3791/58476 30663688

[B27] Dittrich S. Alexi-Meskishvili V. V. Yankah A. C. Dahnert I. Meyer R. Hetzer R. (2000). Comparison of porcine xenografts and homografts for pulmonary valve replacement in children. Ann. Thorac. Surg. 70 (3), 717–722. 10.1016/s0003-4975(00)01532-0 11016299

[B28] Dur O. Yoshida M. Manor P. Mayfield A. Wearden P. D. Morell V. O. (2010). *In vitro* evaluation of right ventricular outflow tract reconstruction with bicuspid valved polytetrafluoroethylene conduit. Artif. organs 34 (11), 1010–1016. 10.1111/j.1525-1594.2010.01136.x 21092044

[B29] Easson G. Laughlin M. Jensen H. Haney K. Girardot M. Jensen M. (2019). Performance changes of venous valves following tissue treatment with novel *in vitro* system. Phlebology. 34 (5), 347–354. 10.1177/0268355518804360 30336758

[B30] Feng Y. Wu Z. S. Hu J. G. Hu T. H. Zhang J. C. Xu Z. J. (2004). [Morphologic and physiochemical properties of bovine jugular conduit stabilized by dye-mediated poto-oxidation]. J. Central South Univ. Med. Sci. 29 (4), 429–431. 16134596

[B31] Fiore A. C. Brown J. W. Turrentine M. W. Ruzmetov M. Huynh D. Hanley S. (2011). A bovine jugular vein conduit: A ten-year bi-institutional experience. Ann. Thorac. Surg. 92 (1), 183–192. 10.1016/j.athoracsur.2011.02.073 21549348

[B32] Fiore A. C. Ruzmetov M. Huynh D. Hanley S. Rodefeld M. D. Turrentine M. W. (2010). Comparison of bovine jugular vein with pulmonary homograft conduits in children less than 2 years of age. Eur. J. Cardio-Thoracic Surg. 38 (3), 318–325. 10.1016/j.ejcts.2010.01.063 20356755

[B33] Forbess J. M. Shah A. S. St. Louis J. D. Jaggers J. J. Ungerleider R. M. (2001). Cryopreserved homografts in the pulmonary position: Determinants of durability. Ann. Thorac. Surg. 71 (1), 54–59. 10.1016/s0003-4975(00)01788-4 11216810

[B34] Gallyamov M. O. Chaschin I. S. Khokhlova M. A. Grigorev T. E. Bakuleva N. P. Lyutova I. G. (2014). Collagen tissue treated with chitosan solutions in carbonic acid for improved biological prosthetic heart valves. Mater. Sci. Eng. C 37, 127–140. 10.1016/j.msec.2014.01.017 24582232

[B35] Gist K. M. Mitchell M. B. Jaggers J. Campbell D. N. Yu J. A. Landeck B. F. (2012). Assessment of the relationship between Contegra conduit size and early valvar insufficiency. Ann. Thorac. Surg. 93 (3), 856–861. 10.1016/j.athoracsur.2011.10.057 22300627

[B36] Göber V. Berdat P. Pavlovic M. Pfammatter J. P. Carrel T. P. (2005). Adverse mid-term outcome following RVOT reconstruction using the Contegra valved bovine jugular vein. Ann. Thorac. Surg. 79 (2), 625–631. 10.1016/j.athoracsur.2004.07.085 15680848

[B37] Guhathakurta S. Galla S. Ramesh B. Venugopal J. R. Ramakrishna S. Cherian K. M. (2011). Nanofiber-reinforced biological conduit in cardiac surgery: Preliminary report. Asian cardiovasc. Thorac. Ann. 19 (3-4), 207–212. 10.1177/0218492311411315 21885543

[B38] Haas N. A. Bach S. Vcasna R. Laser K. T. Sandica E. Blanz U. (2018). The risk of bacterial endocarditis after percutaneous and surgical biological pulmonary valve implantation. Int. J. Cardiol. 268, 55–60. 10.1016/j.ijcard.2018.04.138 30041803

[B39] Hata C. Wang E. Noishiki Y. Tomizawa Y. Tu R. Sung H. W. (1992). Evaluation of a polyepoxy compound fixed biological vascular prosthesis and an expanded polytetrafluoroethylene vascular graft. Artif. Organs 16 (3), 263–266. 10.1111/j.1525-1594.1992.tb00307.x 10078256

[B40] Hellberg K. Ruschewski W. De Vivie E. (1981). Early stenosis and calcification of glutaraldehyde-preserved porcine xenografts in children. Thorac. Cardiovasc. Surg. 29 (06), 369–374. 10.1055/s-2007-1023515 6179219

[B41] Herrmann J. L. Brown J. W. (2020). Seven decades of valved right ventricular outflow tract reconstruction: The most common heart procedure in children. J. Thorac. Cardiovasc. Surg. 160 (5), 1284–1288. 10.1016/j.jtcvs.2020.04.137 32690409

[B42] Hirai K. Baba K. Goto T. Ousaka D. Kondo M. Eitoku T. (2021). Outcomes of right ventricular outflow tract reconstruction in children: Retrospective comparison between bovine jugular vein and expanded polytetrafluoroethylene conduits. Pediatr. Cardiol. 42 (1), 100–108. 10.1007/s00246-020-02458-0 32968822

[B43] Hoashi T. Ichikawa H. Hirose K. Horio N. Sakurai T. Matsuhisa H. (2021). Mid-term outcomes of contegra implantation for the reconstruction of the right ventricular outflow tract to proximal branch pulmonary arteries: Japan multicentre study. Interact. Cardiovasc. Thorac. Surg. 33 (2), 227–236. 10.1093/icvts/ivab075 33755119 PMC8691546

[B44] Huang H. Y. S. Lu J. (2017). Biaxial mechanical properties of bovine jugular venous valve leaflet tissues. Biomech. Model. Mechanobiol. 16 (6), 1911–1923. 10.1007/s10237-017-0927-1 28631145

[B45] Iacobazzi D. Rapetto F. Albertario A. Swim M. M. Narayan S. Skeffington K. (2021). Wharton's jelly-mesenchymal stem cell-engineered conduit for pediatric translation in heart defect. Tissue Eng. Part A 27 (3-4), 201–213. 10.1089/ten.tea.2020.0088 32571164

[B46] Iso (2021). ISO 5840 : 2021 cardiovascular implants - cardiac valve prostheses.

[B47] Iwasaki K. Umezu M. Iijima K. Imachi K. (2002). Implications for the establishment of accelerated fatigue test protocols for prosthetic heart valves. Artif. organs 26 (5), 420–429. 10.1046/j.1525-1594.2002.06956.x 12000438

[B48] Jalal Z. Galmiche L. Lebeaux D. Villemain O. Brugada G. Patel M. (2015). Selective propensity of bovine jugular vein material to bacterial adhesions: An *in-vitro* study. Int. J. Cardiol. 198, 201–205. 10.1016/j.ijcard.2015.07.004 26173058

[B49] Jalal Z. Villemain O. Thambo J. B. Boudjemline Y. (2018). What matters more in testing bacterial adhesion: Flow conditions or choice of bacterial strain? J. Thorac. Cardiovasc. Surg. 156 (2), 737. 10.1016/j.jtcvs.2018.03.085 29730111

[B50] Jiang Z. Wu Z. Deng D. Li J. Qi X. Song M. (2022). Improved cytocompatibility and reduced calcification of glutaraldehyde-crosslinked bovine pericardium by modification with glutathione. Front. Bioeng. Biotechnol. 10, 844010. 10.3389/fbioe.2022.844010 35662844 PMC9160462

[B51] Kadner A. Dave H. Stallmach T. Turina M. Pretre R. (2004). Formation of a stenotic fibrotic membrane at the distal anastomosis of bovine jugular vein grafts (Contegra) after right ventricular outflow tract reconstruction. J. Thorac. Cardiovasc. Surg. 127 (1), 285–286. 10.1016/j.jtcvs.2003.08.031 14752451

[B52] Karamlou T. Blackstone E. H. Hawkins J. A. Jacobs M. L. Kanter K. R. Brown J. W. (2006). Can pulmonary conduit dysfunction and failure be reduced in infants and children less than age 2 years at initial implantation? J. Thorac. Cardiovasc. Surg. 132 (4), 829–838.e5. 10.1016/j.jtcvs.2006.06.034 17000294

[B53] Karamlou T. Ungerleider R. Alsoufi B. Burch G. Silberbach M. Reller M. (2005). Oversizing pulmonary homograft conduits does not significantly decrease allograft failure in children. Eur. J. Cardio-Thoracic Surg. 27 (4), 548–553. 10.1016/j.ejcts.2004.12.054 15784349

[B54] Keane T. J. Swinehart I. T. Badylak S. F. (2015). Methods of tissue decellularization used for preparation of biologic scaffolds and *in vivo* relevance. Methods 84, 25–34. 10.1016/j.ymeth.2015.03.005 25791470

[B55] Kido T. Hoashi T. Kagisaki K. Fujiyoshi T. Kitano M. Kurosaki K. (2016). Early clinical outcomes of right ventricular outflow tract reconstruction with small caliber bovine jugular vein conduit (Contegra®) in small children. J. Artif. Organs 19 (4), 364–371. 10.1007/s10047-016-0908-7 27236437

[B56] Kim K. M. (2001). Cellular mechanism of calcification and its prevention in glutaraldehyde treated vascular tissue. Z. Für. Kardiologie 90 (3), 99–105. 10.1007/s003920170050 11374041

[B57] Knirsch W. Nadal D. (2011). Infective endocarditis in congenital heart disease. Eur. J. Pediatr. 170 (9), 1111–1127. 10.1007/s00431-011-1520-8 21773669

[B58] Krasilnikova A. A. Sergeevichev D. S. Fomenko V. V. Korobeynikov A. A. Vasilyeva M. B. Yunoshev A. S. (2018). Globular chitosan treatment of bovine jugular veins: Evidence of anticalcification efficacy in the subcutaneous rat model. Cardiovasc. Pathol. 32, 1–7. 10.1016/j.carpath.2017.08.003 29049912

[B59] Kueh M. (2020). “Redesign and quantitative assessment of an accelerated venous valve fatigue apparatus,” in Biomedical engineering (Fayetteville, Arkansas: University of Arkansas).

[B60] Leeten K. Ditkowski B. Jashari R. Mela P. Jones E. A. Heying R. (2021). An *in vitro* model to study endothelialization of cardiac graft tissues under flow. Tissue Eng. Part C. Methods 27 (4), 233–241. 10.1089/ten.tec.2020.0359 33544046

[B61] Lichtenberg A. Tudorache I. Cebotari S. Ringes-Lichtenberg S. Sturz G. Hoeffler K. (2006). *In vitro* re-endothelialization of detergent decellularized heart valves under simulated physiological dynamic conditions. Biomaterials 27 (23), 4221–4229. 10.1016/j.biomaterials.2006.03.047 16620956

[B62] Liu W. Hu J. Wu Z. Hu Y. (2009). Research on endothelial cells' attachment and growth on the decellularized bovine jugular vessel circular. Prog. Mod. Biomed. 9 (9), 1616–1619.

[B63] Lu W. Wu Z. s. Hu T. h. Zhang M. Hu Y. r. Jiang Z. b. (2007). Effect of decellular treatment on the framework of extracellular matrix in bovine jugular vein conduit. J. Central South Univ. Med. Sci. 32 (5), 819–823. 18007077

[B64] Lueth E. T. Gist K. M. Burkett D. A. Landeck B. F. Brinton J. T. Meier M. R. (2019). Retrospective comparison of the supported and unsupported bovine jugular vein conduit in children. Ann. Thorac. Surg. 108 (2), 567–573. 10.1016/j.athoracsur.2019.03.021 30974093

[B65] Malekzadeh-Milani S. Ladouceur M. Iserin L. Bonnet D. Boudjemline Y. (2014). Incidence and outcomes of right-sided endocarditis in patients with congenital heart disease after surgical or transcatheter pulmonary valve implantation. J. Thorac. Cardiovasc. Surg. 148 (5), 2253–2259. 10.1016/j.jtcvs.2014.07.097 25218536

[B66] Malone J. M. Brendel K. Duhamel R. C. Reinert R. L. (1984). Detergent-extracted small-diameter vascular prostheses. J. Vasc. Surg. 1 (1), avs0010181–191. 10.1067/mva.1984.avs0010181 6481863

[B67] Mery C. M. (2018). Elucidating the mechanisms of infective endocarditis in bovine jugular vein conduits: Are we any closer? J. Thorac. Cardiovasc. Surg. 156 (2), 739–740. 10.1016/j.jtcvs.2018.04.024 30011767

[B68] Mery C. M. Guzman-Pruneda F. A. De Leon L. E. Zhang W. Terwelp M. D. Bocchini C. E. (2016). Risk factors for development of endocarditis and reintervention in patients undergoing right ventricle to pulmonary artery valved conduit placement. J. Thorac. Cardiovasc. Surg. 151 (2), 432–441. e2. 10.1016/j.jtcvs.2015.10.069 26670191

[B69] Meuris B. De Praetere H. Strasly M. Trabucco P. Lai J. C. Verbrugghe P. (2018). A novel tissue treatment to reduce mineralization of bovine pericardial heart valves. J. Thorac. Cardiovasc. Surg. 156 (1), 197–206. 10.1016/j.jtcvs.2018.01.099 29572021

[B70] Meyns B. Vangarsse L. Boshoff D. Eyskens B. Mertens L. Gewillig M. (2004). The Contegra conduit in the right ventricular outflow tract induces supravalvular stenosis. J. Thorac. Cardiovasc. Surg. 128 (6), 834–840. 10.1016/s0022-5223(04)01173-0 15573067

[B71] Morales D. L. Braud B. E. Gunter K. S. Carberry K. E. Arrington K. A. Heinle J. S. (2006). Encouraging results for the Contegra conduit in the problematic right ventricle–to–pulmonary artery connection. J. Thorac. Cardiovasc. Surg. 132 (3), 665–671. 10.1016/j.jtcvs.2006.03.061 16935124

[B72] Morray B. H. McElhinney D. B. Boudjemline Y. Gewillig M. Kim D. W. Grant E. K. (2017). Multicenter experience evaluating transcatheter pulmonary valve replacement in bovine jugular vein (Contegra) right ventricle to pulmonary artery conduits. Circ. Cardiovasc. Interv. 10 (6), e004914. 10.1161/circinterventions.116.004914 28600328

[B73] Naso F. Gandaglia A. (2018). Different approaches to heart valve decellularization: A comprehensive overview of the past 30 years. Xenotransplantation 25 (1), e12354. 10.1111/xen.12354 29057501

[B74] Nichay N. R. Zhuravleva I. Y. Kulyabin Y. Y. Timchenko T. P. Voitov A. V. Kuznetsova E. V. (2018). In search of the best xenogeneic material for a paediatric conduit: An analysis of clinical data. Interact. Cardiovasc. Thorac. Surg. 27 (1), 34–41. 10.1093/icvts/ivy029 29452369

[B75] Noishiki Y. Hata C. Tu R. Shen S. Lin D. Sung H. (1993). Development and evaluation of a pliable biological valved conduit. Part I: Preparation, biochemical properties, and histological findings. Int. J. Artif. Organs 16 (4), 192–198. 10.1177/039139889301600405 8325696

[B76] Park C. S. Kim Y. J. Lee J. R. Lim H. G. Chang J. E. Jeong S. (2017). Anticalcification effect of a combination of decellularization, organic solvents and amino acid detoxification on glutaraldehyde-fixed xenopericardial heart valves in a large-animal long-term circulatory model. Interact. Cardiovasc. Thorac. Surg. 25 (3), 391–399. 10.1093/icvts/ivx131 28505294

[B77] Patel P. M. Tan C. Srivastava N. Herrmann J. L. Rodefeld M. D. Turrentine M. W. (2018). Bovine jugular vein conduit: A mid-to long-term institutional review. World J. Pediatr. Congenit. Heart Surg. 9 (5), 489–495. 10.1177/2150135118779356 30157735

[B78] Peivandi A. D. Seiler M. Mueller K. M. Martens S. Malec E. Asfour B. (2019). Elastica degeneration and intimal hyperplasia lead to Contegra® conduit failure. Eur. J. Cardio-Thoracic Surg. 56 (6), 1154–1161. 10.1093/ejcts/ezz199 31280306

[B79] Pennel T. Zilla P. (2020). Clinical applications and limitations of vascular grafts. Tissue-Engineered Vasc. Grafts 2020, 3–34.

[B80] Prior N. Alphonso N. Arnold P. Peart I. Thorburn K. Venugopal P. (2011). Bovine jugular vein valved conduit: Up to 10 years follow-up. J. Thorac. Cardiovasc. Surg. 141 (4), 983–987. 10.1016/j.jtcvs.2010.08.037 20884023

[B81] Protopapas A. D. Athanasiou T. (2008). Contegra conduit for reconstruction of the right ventricular outflow tract: A review of published early and mid-time results. J. Cardiothorac. Surg. 3 (1), 62. 10.1186/1749-8090-3-62 19017382 PMC2596120

[B82] Qian T. Wu Z. S. Hu J. G. Yang Y. F. Wu Q. Lu T. (2021). Midterm outcomes of crosslinked acellular bovine jugular vein conduit for right ventricular outflow tract reconstruction. Front. Pediatr. 9, 725030. 10.3389/fped.2021.725030 34485205 PMC8416030

[B83] Qian T. Yuan H. Chen C. Liu Y. Lu T. Huang C. (2021). Conduits for right ventricular outflow tract reconstruction in infants and young children. Front. Surg. 8, 719840. 10.3389/fsurg.2021.719840 34631780 PMC8492946

[B84] Raghav V. Okafor I. Quach M. Dang L. Marquez S. Yoganathan A. P. (2016). Long-term durability of carpentier-edwards magna ease valve: A one billion cycle *in vitro* study. Ann. Thorac. Surg. 101 (5), 1759–1765. 10.1016/j.athoracsur.2015.10.069 26806168

[B85] Rapetto F. Caputo M. Angelini G. (2021). Surgical reconstruction of the right ventricular outflow tract--The clock is still ticking. J. Card. Surg. 36, 3153. 10.1111/jocs.15685 34057241

[B86] Rapetto F. Iacobazzi D. Narayan S. A. Skeffington K. Salih T. Mostafa S. (2022). Wharton’s jelly–mesenchymal stem cell–engineered conduit for pulmonary artery reconstruction in growing piglets. JACC Basic Transl. Sci. 7 (3-1), 207–219. 10.1016/j.jacbts.2021.11.013 35411313 PMC8993765

[B87] Rastan A. J. Walther T. Daehnert I. Hambsch J. Mohr F. W. Janousek J. (2006). Bovine jugular vein conduit for right ventricular outflow tract reconstruction: Evaluation of risk factors for mid-term outcome. Ann. Thorac. Surg. 82 (4), 1308–1315. 10.1016/j.athoracsur.2006.04.071 16996925

[B88] Rittgers S. E. Oberdier M. T. Pottala S. (2007). Physiologically-based testing system for the mechanical characterization of prosthetic vein valves. Biomed. Eng. OnLine 6 (1), 29. 10.1186/1475-925x-6-29 17629916 PMC1947988

[B89] Ross D. (1966). Correction of pulmonary atresia with a homograft aortic valve. Lancet 2, 1446–1447. 10.1016/s0140-6736(66)90600-3 4163445

[B90] Ruzmetov M. Shah J. J. Geiss D. M. Fortuna R. S. (2012). Decellularized versus standard cryopreserved valve allografts for right ventricular outflow tract reconstruction: A single-institution comparison. J. Thorac. Cardiovasc. Surg. 143 (3), 543–549. 10.1016/j.jtcvs.2011.12.032 22340029

[B91] Salem A. M. (2016). Right ventricle to pulmonary artery connection: Evolution and current alternatives. J. Egypt. Soc. Cardio-Thoracic Surg. 24 (1), 47–57. 10.1016/j.jescts.2016.04.009

[B92] Sandica E. Goerg R. Haas N. Laser K. Kececioglu D. Bertram H. (2016). Bovine jugular veins versus homografts in the pulmonary position: An analysis across two centers and 711 patients—conventional comparisons and time status graphs as a new approach. Thorac. Cardiovasc. Surg. 64 (01), 025–035. 10.1055/s-0035-1554962 26322831

[B93] Sawada K. Terada D. Yamaoka T. Kitamura S. Fujisato T. (2008). Cell removal with supercritical carbon dioxide for acellular artificial tissue. J. Chem. Technol. Biotechnol. 83 (6), 943–949. 10.1002/jctb.1899

[B94] Schoen F. J. Levy R. J. (2005). Calcification of tissue heart valve substitutes: Progress toward understanding and prevention. Ann. Thorac. Surg. 79 (3), 1072–1080. 10.1016/j.athoracsur.2004.06.033 15734452

[B95] Senage T. Paul A. Le Tourneau T. Fellah-Hebia I. Vadori M. Bashir S. (2022). The role of antibody responses against glycans in bioprosthetic heart valve calcification and deterioration. Nat. Med. 28 (2), 283–294. 10.1038/s41591-022-01682-w 35177855 PMC8863575

[B96] Sersar S. I. Sallam A. Hassan M. Abdelzaher M. (2014). Three indications for bovine jugular vein grafts in a single tertiary centre in jeddah: A retrospective study. J. Cardiol. Ther. 2014 (2), 27–31. 10.12970/2311-052x.2014.02.01.6

[B97] Sharma A. Cote A. T. Hosking M. C. Harris K. C. (2017). A systematic review of infective endocarditis in patients with bovine jugular vein valves compared with other valve types. JACC Cardiovasc. Interv. 10 (14), 1449–1458. 10.1016/j.jcin.2017.04.025 28728659

[B98] Shebani S. O. Mcguirk S. Baghai M. Stickley J. Degiovanni J. Bulock F. (2006). Right ventricular outflow tract reconstruction using Contegra® valved conduit: Natural history and conduit performance under pressure. Eur. J. cardio-thoracic Surg. 29 (3), 397–405. 10.1016/j.ejcts.2005.11.040 16439155

[B99] Tao Y. Zhongshi W. Hu Y. Chunlin W. Tiehui H. Tan Q. (2012). Heparin nanomodification improves biocompatibility and biomechanical stability of decellularized vascular scaffolds. Int. J. Nanomedicine 7, 5847–5858. 10.2147/ijn.s37113 23226016 PMC3512543

[B100] Teebken O. Puschmann C. Aper T. Haverich A. Mertsching H. (2003). Tissue-engineered bioprosthetic venous valve: A long-term study in sheep. Eur. J. Vasc. endovascular Surg. 25 (4), 305–312. 10.1053/ejvs.2002.1873 12651167

[B101] Tod T. J. Dove J. S. (2016). The association of bound aldehyde content with bioprosthetic tissue calcification. J. Mat. Sci. Mat. Med. 27 (1), 8. 10.1007/s10856-015-5623-z 26610931

[B102] Tomizawa Y. Noishiki Y. Okoshi T. Koyanagi H. (1988). Development of a small-caliber vascular graft with antithrombogenicity induced by extreme hydrophilicity. ASAIO Trans. 34 (3), 644–650. 3143386

[B103] Tweddell J. S. Pelech A. N. Frommelt P. C. Mussatto K. A. Wyman J. D. Fedderly R. T. (2000). Factors affecting longevity of homograft valves used in right ventricular outflow tract reconstruction for congenital heart disease. Circulation 102 (3), III130–III135. 10.1161/01.cir.102.suppl_3.iii-130 11082375

[B104] Ugaki S. Rutledge J. Aklabi M. A. Ross D. B. Adatia I. Rebeyka I. M. (2015). An increased incidence of conduit endocarditis in patients receiving bovine jugular vein grafts compared to cryopreserved homograft for right ventricular outflow reconstruction. Ann. Thorac. Surg. 99 (1), 140–146. 10.1016/j.athoracsur.2014.08.034 25440268

[B105] Veloso T. R. Claes J. Van Kerckhoven S. Ditkowski B. Hurtado-Aguilar L. G. Jockenhoevel S. (2018). Bacterial adherence to graft tissues in static and flow conditions. J. Thorac. Cardiovasc. Surg. 155 (1), 325–332. e4. 10.1016/j.jtcvs.2017.06.014 28712577

[B106] Veloso T. R. Ditkowski B. Mela P. Hoylaerts M. F. Heying R. (2018). Are plasma proteins key players in the pathogenesis of infective endocarditis? J. Thorac. Cardiovasc. Surg. 156 (2), 738–739. 10.1016/j.jtcvs.2018.04.053 30011766

[B107] Vesely I. Boughner D. R. Leeson-Dietrich J. (1995). Bioprosthetic valve tissue viscoelasticity: Implications on accelerated pulse duplicator testing. Ann. Thorac. Surg. 60, S379–S383. 10.1016/0003-4975(95)00261-i 7646192

[B108] Wang E.-S. Fan X. S. Xiang L. Li S. J. Zhang H. (2018). Surgical outcome after complete repair of tetralogy of Fallot with absent pulmonary valve: Comparison between bovine jugular vein-valved conduit and monocusp-valve patch. World J. Pediatr. 14 (5), 510–519. 10.1007/s12519-018-0169-z 30062647

[B109] Wang H. Hu J. Wu Z. Yang J. Hu T. Deng Y., et al. (2005). Immunological study of transplanted bovine jugular vein conduits treated by different crosslinking methods. J. Chin. Physician 7 (7), 928–930.

[B110] Wells W. J. Arroyo H. Bremner R. M. Wood J. Starnes V. A. (2002). Homograft conduit failure in infants is not due to somatic outgrowth. J. Thorac. Cardiovasc. Surg. 124 (1), 88–96. 10.1067/mtc.2002.121158 12091813

[B111] Wojtalik M. Mrowczynski W. Eromski J. Bartkowski R. (2003). Does contegra xenograft implantation evoke cellular immunity in children? Interact. Cardiovasc. Thorac. Surg. 2 (3), 273–278. 10.1016/s1569-9293(03)00058-6 17670046

[B112] Xiling Z. Puehler T. Seiler J. Gorb S. N. Sathananthan J. Sellers S. (2022). Tissue engineered transcatheter pulmonary valved stent implantation: Current state and future prospect. Int. J. Mol. Sci. 23 (2), 723. 10.3390/ijms23020723 35054905 PMC8776029

[B113] Xu Z. Song L. Yang J. Cheng Y. Huang W. (2012). Research of the acellular valved bovine jugular vein conduit treated with proanthocyanidin. Prog. Mod. Biomed. 12 (30), 5831–5835.

[B114] Yamamoto Y. Yamagishi M. Miyazaki T. (2015). Current status of right ventricular outflow tract reconstruction: Complete translation of a review article originally published in kyobu geka 2014; 67: 65–77. Gen. Thorac. Cardiovasc. Surg. 63 (3), 131–141. 10.1007/s11748-014-0500-0 25503561

[B115] Yoldaş T. Örün U. A. Tak S. (2019). True aneurysmal dilatation of a valved bovine jugular vein conduit after right ventricular outflow tract reconstruction: A rare complication. Cardiol. Young 29 (8), 1097–1098. 10.1017/s1047951119001422 31280726

[B116] Yong M. S. Yim D. d'Udekem Y. Brizard C. P. Robertson T. Galati J. C. (2015). Medium-term outcomes of bovine jugular vein graft and homograft conduits in children. ANZ J. Surg. 85 (5), 381–385. 10.1111/ans.13018 25708132

[B117] Yoshinaga M. Niwa K. Niwa A. Ishiwada N. Takahashi H. Echigo S. (2008). Risk factors for in-hospital mortality during infective endocarditis in patients with congenital heart disease. Am. J. Cardiol. 101 (1), 114–118. 10.1016/j.amjcard.2007.07.054 18157976

[B118] Yuan J. M. Xiong S. H. Liu Z. Wen Y. Dang R. S. Shen M. R. (2016). Functional analysis *in vivo* of engineered valved venous conduit with decellularized matrix and two bone marrow-derived progenitors in sheep. J. Tissue Eng. Regen. Med. 10 (7), 554–563. 10.1002/term.1748 23904287

[B119] Zhang H.-F. Chen G. Ye M. Yan X. G. Tao Q. L. Jia B. (2017). Mid-to long-term outcomes of bovine jugular vein conduit implantation in Chinese children. J. Thorac. Dis. 9 (5), 1234–1239. 10.21037/jtd.2017.05.02 28616273 PMC5465117

[B120] Zhuravleva I. Y. Dokuchaeva A. A. Karpova E. V. Timchenko T. P. Titov A. T. Shatskaya S. S. (2021). Immobilized bisphosphonates as potential inhibitors of bioprosthetic calcification: Effects on various xenogeneic cardiovascular tissues. Biomedicines 10 (1), 65. 10.3390/biomedicines10010065 35052745 PMC8773418

[B121] Zhuravleva I. Y. Karpova E. V. Dokuchaeva A. A. Kuznetsova E. V. Vladimirov S. V. Ksenofontov A. L. (2022). Bovine jugular vein conduit: What affects its elastomechanical properties and thermostability? J. Biomed. Mat. Res. A 110 (2), 394–408. 10.1002/jbm.a.37296 34390309

[B122] Zhuravleva I. Y. Nichay N. R. Kulyabin Y. Y. Timchenko T. P. Korobeinikov A. A. Polienko Y. F. (2018). In search of the best xenogeneic material for a paediatric conduit: An experimental study. Interact. Cardiovasc. Thorac. Surg. 26 (5), 738–744. 10.1093/icvts/ivx445 29346675

[B123] Zou M.-H. Ma L. Cui Y. Q. Wang H. Z. Li W. L. Li J. (2021). Outcomes after repair of pulmonary atresia with ventricular septal defect and major aortopulmonary collateral arteries: A tailored approach in a developing setting. Front. Cardiovasc. Med. 8, 665038. 10.3389/fcvm.2021.665038 33937364 PMC8079636

